# Photoperiodic history modulates the response of the saccus vasculosus transcriptome to seawater exposure in Atlantic salmon

**DOI:** 10.1007/s00359-025-01779-w

**Published:** 2025-11-06

**Authors:** Alexander C. West, Shona H. Wood, Marianne Iversen, Mattis Jayme van Dalum, Even H. Jørgensen, Simen R. Sandve, David G. Hazlerigg

**Affiliations:** 1 Arctic Seasonal Timekeeping Initiative (ASTI), Department of Arctic and Marine Biology, University of Tromsø, Tromsø, Norway; 2 Department of Animal and Aquacultural Sciences (IHA), Faculty of Life Sciences (BIOVIT), Norwegian University of Life Sciences (NMBU), Ås, Norway

**Keywords:** Atlantic salmon, *Salmo salar*, Saccus vasculosus, Smoltification, Photoperiod, Seasonal

## Abstract

Atlantic salmon (*Salmo salar*) move from fresh- to seawater environments following a seasonally timed preparative transition called smoltification, which takes place under photoperiodic control in the freshwater environment. In masu salmon (*Oncorhynchus masou*), coordination of photoperiodic sexual maturation is proposed to involve in a fish-specific circumventricular organ, the saccus vasculosus (SV), through its intrinsic opsin-based light sensitivity, thyrotrophin secretion and modulation of deiodinase activity (TSH-DIO cascade). The saccus vasculosus is a highly vascularized structure located on the ventral side of the hypothalamus and its interface between the blood and cerebrospinal fluid also hints at a role in ionic balance of the cerebrospinal fluid (CSF). Both the potential photoperiodic and ionic functions of the SV led us to perform transcriptome analysis of the SV in smoltification in Atlantic salmon. Specifically, we compared transcriptomes of SVs collected from freshwater fish following exposure to an 8-week stimulated winter photoperiod followed by 8-week simulated summer photoperiod, or a 16-week simulated winter photoperiod control and from both photoperiod treatments after 24 h exposure to seawater. Our data show that SV response to seawater exposure is highly dependent on photoperiodic history and identifies ependymin as a major secretory output of the SV, consistent with a role in control of CSF composition. Conversely, we could not detect crucial elements of the opsin-TSH-DIO cascade suggesting that the photoperiodic history-dependence of the SV to seawater exposure is unlikely to stem from SV-intrinsic responses to photoperiod.

**Supplementary Information:**

The online version contains supplementary material available at 10.1007/s00359-025-01779-w.

## Introduction

Annual changes in day-length (photoperiod) alter the seasonal environment, particularly at higher latitudes. As a result, many organisms compartmentalize elements of their life history to specific times of year (Hazlerigg et al. 2023). Atlantic salmon (*Salmo salar*) undergo two major seasonally timed developmental processes in their lifecycle: reproduction and smoltification (Mobley et al. 2021; West et al. 2020a). Reproduction occurs following migration to their natal streams in the autumn (Mobley et al. 2021). Smoltification occurs earlier in life when immature ‘parr’ salmon prepare to leave their freshwater environments and migrate to the ocean during the spring. While smoltification is a complex event that includes changes in physical appearance, growth, endocrinology and immune function (Johansson et al. 2016; Nuñez-Ortiz et al. 2018; West et al. 2021), its most striking feature is the change in osmoregulatory function which allows smolts to thrive under the osmotic gradient of their seawater environment (Stefansson et al. 2008).

**Fig. 1:**
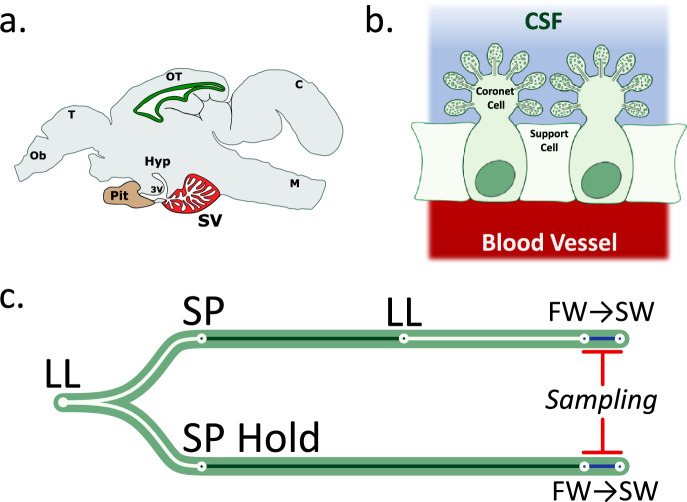
The saccus vasculosus and experimental design **a** Atlantic salmon have a well-developed saccus vasculosus (SV) located next to the ventral side of the brain, caudal to the pituitary gland and connected to the third ventricle. Highlighted in red. Abbreviations: Ob. Olfactory bulb, T. Telencephalon, OT. Optic tectum, C. cerebellum, M. mesencephanlon, Pit. Pituitary, 3 V. third ventricle, SV. Saccus vasculosus. **b** The SV contains coronet cells whose protrusions into the cerebrospinal fluid (CSF) are covered in several globule structures. Coronet cells are flanked by supporting cells. **c** Experimental design: Atlantic Salmon were reared under constant light (LL), then transferred to short photoperiod (SP). One group was held on SP for the rest of the experiment (bottom – SP Hold, 16 weeks). The other group were transferred to LL for 8 weeks after eight weeks of SP(top—SPLL). Both groups sampled in freshwater (FW) and after 24 h in seawater (SW)

Atlantic salmon, like other vertebrates, time seasonal changes in phenotype by integrating photoperiod information (Hazlerigg et al. 2023). Short photoperiods (e.g. 8L:16D) provide a winter signal and long photoperiods (e.g. 18L:6D; or constant light, LL) provide a summer signal. Current working models for the physiological mechanism underlying the vertebrate physiological response focus on thyrotrophin signaling and hypothalamic changes in thyroid hormone (TH) metabolism (Hanon et al. 2008; Masumoto et al. 2010; Nakao et al. 2008). In photoperiodic birds and mammals, decoding of photoperiod takes place in the pars tuberalis (PT), a specialized pituitary tissue (West and Wood 2018). On sensing summer-like daylengths, the PT rapidly produces a modified form of thyrotrophin (TSH) whose retrograde action in hypothalamus stimulates the expression of deiodinase type II (*dio2*), a thyroid hormone activating enzyme, within specialized tanycyte cells lining the third ventricle. The induction of DIO2 and concomitant suppression of DIO3, a thyroid hormone inactivating enzyme, leads to the conversion of the biologically inert T4 form of thyroid hormone to the bioactive variant T3. Local T3 elevation within the hypothalamus then orchestrates a neuroendocrine program that stimulates organism-specific, summer-type physiological states. In contrast, short winter-like photoperiods lead to low levels of PT-type TSH and DIO2, and increased DIO3 levels reduce the hypothalamic T3 concentration which is linked to winter-type physiological states (Hanon et al. 2008; Masumoto et al. 2010; Nakao et al. 2008; Yoshimura et al. 2003). While teleosts do not have an anatomically distinct PT (Trudeau and Somoza 2020), two models nonetheless place seasonally dependent changes in TSH expression at the core of the photoperiod integration mechanism. Based on studies in masu salmon, Yoshimura and colleagues have described the presence of components of the TSH-DIO pathway, including light-sensitive opsins, in coronet cells of the saccus vasculosus (SV), a fish-specific circumventricular organ (Fig. 1a–b) (Nakane et al. 2013). In addition, they reported that surgical removal of the SV in masu salmon (*Oncorhynchus masou*) blocks winter photoperiod-induced testicular growth, suggesting that the SV may be a seasonal sensor of photoperiod and coordinator of seasonal reproduction in salmonids (Nakane et al. 2013). Contrastingly, in Atlantic salmon, in has been reported that a novel *tshβ* subunit isoform, designated *tshβb* is expressed in the dorsal region of the pars distalis, with levels increasing markedly during photoperiod-dependent smoltification, leading to the proposal that this isoform plays an analogous role to PT-derived TSH in birds and mammals (Fleming et al. 2019; Irachi et al. 2021).

To date, no studies have investigated the relationship between the SV and smoltification. It is notable, however, that the SV coronet cell globules of several teleosts, including rainbow trout (*Oncorhynchus mykiss*), contain high densities of Ca^2+^ containing vesicles which are suggested to act as reservoirs that release calcium ions into the cerebrospinal fluid (CSF) of the third ventricle (Jansen et al. 1981, 1982; Graña et al. 2012; Cid et al. 2015). The SV is also extensively innervated by neurons that project both to and from the hypothalamus and neurosecretory CSF contacting dopaminergic neurons (Joy and Sathyanesan 1979; Sueiro et al. 2007). While there are no categorical divisions between teleosts from different ecological niches, there is a notable loss or reduction of the SV in most primary freshwater fish contrasting with marine teleosts who usually have well-developed SVs (Tsuneki 1992). The SV of some euryhaline fish changes with seawater exposure. For example, the prevalence of glycosaminoglycans, polysaccharides important for ECM function (Köwitsch et al. 2018), on the cell surface of stickleback (*Gasterosteus aculeatus*) and rainbow trout SV coronet cells is greater when fish are kept on freshwater compared to seawater (Emanuelsson and von Mecklenburg 1972; Benjamin 1974). Interestingly, injection of SV homogenate from seawater-acclimated rainbow trout into the third ventricle of freshwater-acclimated rainbow trout increased their seawater survival time compared to uninjected animals, supporting a role of the SV in SW adaptation (Emanuelsson and von Mecklenburg 1974). Together, these data suggest a role for the SV in CSF ion composition which may be adaptive during marine migration of salmon during smoltification.

To determine if the SV plays a role in photoperiodic control of smoltification and / or osmoregulation, we have conducted an analysis of transcriptomic changes in the SV of Atlantic salmon subjected to a standard photoperiod protocol known to induce smoltification under laboratory conditions. Our data support earlier reports suggesting a role for the SV in calcium homeostasis and indicate that the SV is sensitive to both photoperiod and salinity, with a significant interaction between these factors. Despite this, key components of the TSH-DIO pathway are not expressed in the SV of juvenile Atlantic salmon, implying that these observed changes are secondary to neuroendocrine regulation mediated by other neuroanatomical sites.

## Materials and methods

### Animal welfare statement

All studies were performed in accordance with Norwegian and European legislation on animal research. The smoltification experiment was conducted as part of ongoing smolt production at Havbruksstasjonen i Tromsø, which is approved by the Norwegian Animal Research Authority (NARA) for the containment and experiments on fresh and seawater fish. Formal approval of our experimental protocol was not required because the experimental conditions were consistent with routine animal husbandry practices and therefore there was no compromise of animal welfare.

### Experimental design

Atlantic salmon (*Salmo salar*, Aquagen commercial stain, Norway) were raised from hatching in freshwater under continuous light (> 200 lx at water surface) at ambient temperature (~ 10 °C). Juvenile fish were housed in 500L tanks and fed continuously with pelleted salmon feed (Skretting, Stavanger, Norway). At 11 months of age, 1400 parr (mean weight 40.3 g) were distributed among distributed among eight 300 L circular tanks and allowed to acclimate for one week.

Following acclimation, the fish were treated in two groups. Group 1 (SP-hold) were transferred to short photoperiod (SP; 8 h light/24 h) for 16 weeks. Group 2 (SPLL) were transferred to SP for 8 weeks before transfer to constant light (LL) for 8 weeks. Constant light provides a summer stimulatory photoperiod in Atlantic salmon and is commonly used in aquaculture. Fish were sampled in FW prior to SW transfer, then the remaining fish from both group 1 and group 2 were transferred to a full-strength seawater tank (34 ‰ salinity) under constant light. After 24 h in seawater the final group of fish were collected (see Fig. 1c for experimental design). The experiments were performed in duplicate tanks, five fish were taken from each duplicate tank at each collection point, sex was not determined.

### Tissue collection

On collection, the fish were netted out and lethally anesthetized in benzocaine (150 ppm; Benzoak Vet, ACD Pharma, Norway). Once fish opercula stopped moving, blood was sampled from the caudal vein, then the fish were decapitated and SVs and gill filaments rapidly dissected and stored in RNAlater (Sigma-Aldrich, USA) at 4˚C for 24 h, then frozen at -80˚C until further processing. Additional gill filament samples (2–3 secondary filaments) were collected in 100ul ice cold SEI buffer (0.15 M sucrose, 0.01 M N_2_EDTA, 0.05 M imidazole; Sigma, USA) and frozen on dry ice before transfer to -80˚C for storage (Mccormick 1993). Blood samples were collected in 2 ml lithium-heparinised vacutainers (Beckton Dickinson, Puls Norge, Norway) and stored on ice before centrifugation (6000 × *g*) for 10 min at 4 °C. Plasma supernatents were aliquoted and stored at − 20 °C.

### Blood plasma osmolality measurements

Blood plasma samples were thawed on ice then osmolality measurements were made from 50 µl aliquots using a three-point (0, 300, 850 mOsmol/kg) calibrated osmometer OSMOMAT^®^ 030 (GONOTEC, Berlin, Germany).

### Sodium, potassium ATPase (NKA) activity

NKA assays were performed in 96 well format following (Mccormick 1993). Briefly, gill tissue in SEI buffer was thawed and homogenised in the presence of sodium deoxycholate (0.1%; Sigma, USA). The samples were centrifuged at 3000 × *g* for 30 s then the supernatant assayed for ATP-ADP hydrolysis activity, with and without the NKA activity inhibitor ouabain. Spectrophotometer readings (Spectramax Plus 384, USA) at 340 nm were measured at 25 °C at 30 s intervals for 10 min to derive NKA activity Vmax. NKA activity was normalised to protein concentration measured by bicinchonic acid protein assay kit (Pierce, Thermo Fisher, USA) and expressed in units of ATPase activity per mg protein. NKA activity values are replotted from Iversen et al. (2020) in Figure S1a.

### RNA extraction

SVs were selected from two fish per sampling point from duplicate tanks. SV tissue was disrupted using a TissueLyser II (QIAgen, Germany) and RNA extracted using an RNeasy plus micro kit (QIAgen, Germany). RNA concentrations were measured using a NanoDrop ND2000c spectrophotometer (NanoDrop Technologies, USA) and stored at − 80˚C until further processing. RNA from gill filaments were extracted as detailed in Iversen et al. (2020).

### Quantitative polymerase chain reaction

Gill RNA was processed as in Iversen et al. (2020). and quantitative PCR for *nkaa1b(ii)* and *ctfr* conducted to confirm smolt status, data are replotted in Figure S1a. Primer sequences, reagents and thermal cycler programmes are detailed in Iversen et al. (2020).

### RNA sequencing

Sequencing libraries were generated using TruSeq Stranded mRNA HS kit (Illumina, USA). Library concentration was measured using a QUBIT BR kit (Thermo Scientific, USA) and mean length was measured using a 2100 Bioanalyzer and DNA 1000 kit (Agilent Technologies, USA). Individual samples were then barcoded using Illumina HiSeq indexes and single-end 100 bp sequencing of the samples were performed using an Illumina HiSeq 2500 at the Norwegian Sequencing Centre (University of Oslo, Norway). Cutadapt (v4.0) was next used to remove sequencing adapters, trim low quality bases and remove short sequencing reads under the parameters -q 20 -O 8—minimum-length 40 (v4.0, Martin 2011). FastQC (v0.12.1, Barbraham Institute, UK) was used to perform quality control of the reads. Three samples were excluded from the analysis due to low read counts (< 1 million; Supplementary Table 7). Transcripts from single-end RNAseq data were quantified based on the Atlantic salmon reference transcriptome version 3 (Ssal_v3.1, GCA_905237065.2) by using Salmon (v 1.1.0.) on mapping-mode using the flags –keepDuplicates for indexing. The flags –validateMappings, –gcBias and –numBootstraps 100 were used during quantification (Patro et al. 2017). Transcript level counts were uploaded to EdgeR (3.42.4) using the command catchSalmon (Robinson et al. 2009). Normalised counts were filtered by determining the median counts per million (CPM) across the whole experiment for each transcript and applying a cut off of 0.77CPM, which for our 13 samples sets a threshold of 10 counts per transcript over the entire dataset (Ji and Sadreyev 2018). Differential transcript expression was then calculated by ANOVA-like contrasts, in some cases, we therefore present multiple differentially regulated transcripts that are derived from the same gene (Supplementary Tables 2–6). The final replicate numbers in each group were therefore: SP-hold_FW = 4, SP-hold_SW = 3, SPLL_FW = 2, SPLL_SW = 4. Data is accessible online (GEO accession number GSE291612).

### Gene ontology analysis and transcription factor binding site analysis

Mouse and zebrafish homologues to Atlantic salmon genes were identified using BioMart (Smedley et al. 2009). The target and background sets of genes were then submitted to the GOrilla gene ontology tool along to identify enriched GO terms (Eden et al. 2009). Enrichment for transcription factor binding sites was determined using SalmotifDB (Mulugeta et al. 2019).

## Results

### Photoperiodic stimulation of smoltification

In a parallel study (Iversen et al. 2020), we confirmed smolt status by measuring gill NKA activity and qPCR for two smolt-associated ion channel subunits (*nkaa1b(ii)* and *cftr)*, to infer the preparation of the fish to the osmoregulatory challenges of marine migration (Figure S1a) (Nilsen et al. 2007; Mccormick et al. 2013). Gill NKA activity was increased, as was *nkaa1b(ii)* and *cftr* RNA expression, in fish collected in the SPLL FW group compared to the SP-hold FW group, typical of fish adopting smolt physiology (Figure S1a; Mccormick et al. 2013; Nilsen et al. 2007). We next measured the ability of the fish to hypo-osmoregulate by measuring of blood plasma osmolality and chloride concentrations following 24 h in full strength seawater. Both blood osmolality and chloride levels were increased in the SP-hold group relative to the SPLL group, indicating a reduced capacity of the SP-hold group to defend against the osmotic gradient experienced in seawater (Figure S1b). Together these data show that, consistent with previous work, the photoperiod treatments of our experiment (Fig. 1C) were sufficient to stimulate smoltification in the SPLL group but not in the SP-hold group (Handeland and Stefansson 2001; Strand et al. 2018).

### Transcriptomic analysis of saccus vasculosus highlights role in ependymin production and exocytosis.

We next wanted to examine our RNAseq data for insights into the SV biology of Atlantic salmon. Our SV data identified 3342 transcripts expressed with > 30 transcripts per million reads (TPM; Supplementary Table 1). We analyzed these highly expressed transcripts, by collapsing them by genes and identifying over-representation of genes associated with specific gene ontology (GO) terms (Supplementary Table 1). The most significant over-represented GO terms were associated with translation (e.g. GO:0006412 translation, GO:0003735 structural constituent of ribosome, GO:0005840 ribosome), neurons (e.g. GO:0045202 synapse, GO:0097458 neuron part) and oxygen transport (e.g. GO:0031721 hemoglobin alpha binding, GO:0005344 oxygen carrier activity).

We also further examined highly abundant transcripts with the expectation that their protein products may have a major role in SV function. Ranking the genes by average TPM highlighted *Ependymin-1* (*epd1*) and *Ependymin-2* (*epd2*), calcium-binding extracellular matrix (ECM) proteins (Hoffmann & Schwarz 1996), whose combined transcript abundance represented an impressive 15% of the total counts. Other abundant transcripts included: *parvalbumin* (*prvt*), an intracellular calcium buffer and known SV marker gene (Schwaller 2009; Maeda et al. 2015); *2-peptidylprolyl isomerase A* (*ppia*, aka. *Cyclophilin A*) an enzyme which facilitates protein folding and whose secretion is linked to inflammation (Lang et al. 1987; Nigro et al. 2013); and the oxygen transporter *Hemoglobin* (*hbb*), whose prevalence is likely due to the high vascularization, and therefore increased red blood cell complement, of our SV sample (Chakrabarti and Khatun 2017). We note that there are also several highly expressed genes in our dataset whose function remains uncharacterised (Supplementary Table 1).

### Photoperiod changes the SV transcriptional response to seawater transfer

**Fig. 2:**
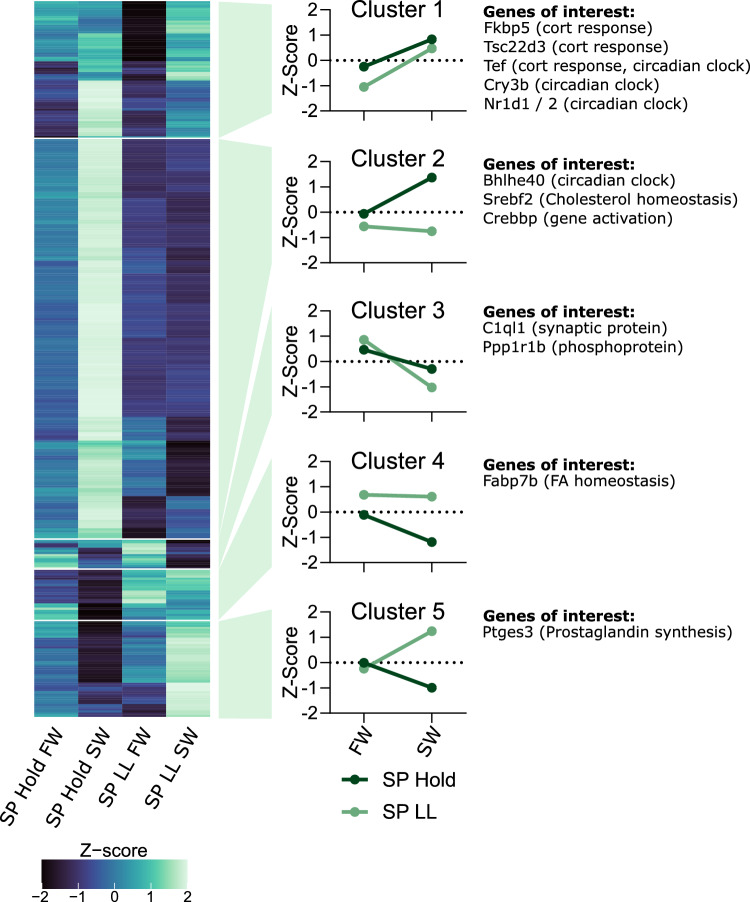
The Atlantic salmon transcriptome is sensitive to both photoperiod and water quality. Heatmap showing the mean expression of differentially expressed genes clustered into five major clusters of gene expression profiles. Specific genes of interest are highlighted next to each clustering group

An ANOVA-like test on SV transcriptomes from SP hold FW, SP hold SW, SPLL FW and SPLL SW groups (Fig. 1c) identified a list of 334 transcripts that were differentially regulated with an FDR cutoff of < 0.01 (Supplementary Tables 2–6). Hierarchical clustering of the groups separated their expression into five major clusters with distinctive expression profiles (Fig. 2).

Cluster 1 and 3 transcript expression were affected by seawater transfer independent of photoperiod (Fig. 2). Cluster 1 transcripts were induced by seawater transfer (64 transcripts; Fig. 2). While there was no significant enrichment of GO terms (FDR cutoff < 0.01) or TFBSs (q-value cutoff < 0.01), the glucocorticoid receptor binding site was enriched (q-value = 0.055) and the group contained several genes with strong links to cortisol response pathways (e.g. Fkbp5; (Grad and Picard 2007), Tsc22d3 (Riccardi 2015)), some of which are also related to circadian clock function (e.g. Tef (Fonjallaz et al. 1996)). Cluster 3 transcripts, in contrast to cluster 1, were suppressed by seawater transfer (14 transcripts; Fig. 2). This cluster includes *c1ql1*, a secreted synaptic protein (Sigoillot et al. 2015), and *ppp1r1b*, which encodes *Protein phosphatase 1 regulatory subunit 1B*, also known as *dopamine- and cAMP-regulated neuronal phosphoprotein 32* (darpp*-32*), which is involved in dopamine and glutamate-dependent synaptic plasticity (Svenningsson et al. 2004).

Cluster 4 was increased under SPLL compared to SP hold and comparatively stable following seawater transfer under both photoperiod treatments (24 transcripts; Fig. 2). The group had no enriched GO terms (FDR cutoff < 0.01), but we identified a significant enrichment of the GMEB1 TFBS, a glucocorticoid modulatory element (Zeng et al. 2000). We note, however, that the expression of *gmeb1* is low (22426th most expressed transcript, average TPM 1.46; supplementary Table 1). 

Cluster 2 and 5, which together make up over two thirds of the differentially regulated transcripts, showed photoperiod-dependence in their response to SW challenge. Cluster 2 transcripts were induced following seawater transfer in the SP hold group, but not in the SPLL group (187 transcripts; Fig. 2). The group was enriched for several GO terms relating to transcriptional regulation (FDR cutoff < 0.01; Supplementary Table 3; Fig. 2), and was enriched for 295 TFBS (q-value cutoff < 0.01), including several immediate early genes (e.g. EGR1, FOS and JUN) and TEF, a PAR ZIP transcription factor linked to the circadian clock and cortisol response in salmon gill tissue (Fonjallaz et al. 1996; Vatine et al. 2009; West et al. 2020b). Notably, cluster 2 had high expression of the transcription regulators *bhlhe40* (aka. *dec1*), a circadian clock repressor linked to photoperiodic timing in mammals (Wood et al. 2015), *huwe1*, a central coordinator of ubiquitination (Kao et al. 2018), and *srebf2*, a transcriptional regulator of genes involved in cholesterol biosynthesis (Madison 2016). Cluster 5 transcripts were suppressed following seawater transfer in the SP hold group and induced by seawater transfer in the SPLL group (45 transcripts; Fig. 2). The cluster was not enriched for GO terms (FDR cutoff < 0.01; Supplementary Table 6) or TFBSs (q-value cutoff < 0.01), but did express high levels of *ptges2*, an enzyme that catalyzes the conversion of prostaglandin to its bioactive E2 form and can influence vascular tone (Hara et al. 2010).

### The TSH-DIO pathway in the SV is incomplete and not consistently regulated by photoperiod or water salinity

**Fig. 3:**
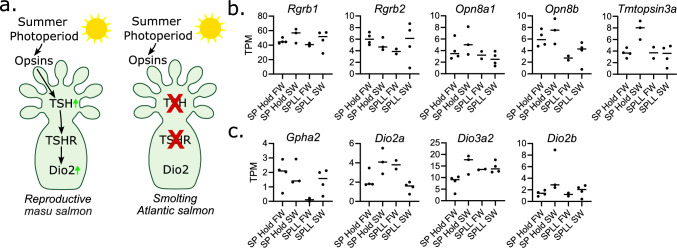
TSH-Dio pathway lacks key elements in the Atlantic salmon saccus vasculosus during parr-smolt transformation **a** schematic showing the pathway from light to increased Dio2 expression identified in precocious male masu salmon (Nakane et al. 2013) and the comparison to the elements identified in our data of the smoltifing Atlantic salmon. **b** Opsin transcript expression in the Atlantic salmon SV. **c** *Gpha2* and deiodinase transcript expression in the Atlantic salmon SV

To determine if the TSH-DIO pathway (Fig. 3a) was present or differentially regulated by photoperiod or water salinity, we extracted counts from each component of the pathway from our transcriptomic dataset. We first checked for the expression of opsins, a family of conserved photosensitive G-protein coupled receptors. Out of the 57 known Atlantic salmon opsins we detected the transcript expression of five: *rgrb1, rgrb2, opn8a1, opn8b and tmtopsin3a* (Fig. 3b, Supplementary Table 8) (Eilertsen et al. 2022a, b). We next checked for the expression of the genes that encode the TSH protein heterodimer and the TSH receptor, TSHR. Atlantic salmon have two *tshβ* gene duplicates (*tshβa* and *tshβb*) (Fleming et al. 2019) and two alpha subunit gene duplicates (*glha1* and *gpha2*), however, of these four genes only *gpha2* was expressed in our samples, at a low abundance (mean < 1.5 TPM) and with no significant change across the experiment (Fig. 3C). *Tshr,* was also absent. We did, however, detect the expression of *dio2* (both duplicate genes *dio2a* and *dio2b*) and *dio3a2* (Fig. 3C), but for none of these transcripts did we see significant effects of photoperiod or SW exposure.

## Discussion

To investigate the potential role of the SV during smoltification and seawater transfer in Atlantic salmon, we have characterised the transcriptomic response of the SV to both photoperiod and seawater transfer. Our transcriptomic analysis identified 334 transcripts in the SV whose expression changed in response to photoperiod and / or salinity changes. Apart from genes in cluster 1, which comprise about a fifth of all DEGs detected, the overall message from this analysis is that salinity-dependent changes in SV gene expression are highly dependent on prior photoperiodic history. In some cases, this means a heightened response in smoltified fish (e.g. cluster 3) and in others an attenuated response (e.g. cluster 4). Potentially, these stem from differences in the systemic physiological response to SW exposure due to smoltification-related preparatory changes in physiology—for example in the hypo-osmoregulatory capacity of the gill, manifested in differences in plasma osmolality and ionic composition (see Figure S1; Hoar 2024). Alternatively, they may reflect photoperiodic programming at the level of the SV itself, which then leads to altered responses to ionic, metabolic or hormonal changes upon exposure to SW. Disentangling these scenarios will be difficult, and it is likely that both phenomena contribute to the observed changes in SV responsiveness. In this regard, it is interesting to note that cluster 1 genes (i.e. genes whose expression is increased by SW exposure, independent of photoperiodic history / smoltification status) include a group of circadian clock genes (*cry1*, *tef, nr1d1* and *nr1d2),* which our previous work shows are also induced by seawater transfer in the Atlantic salmon gill (West et al. 2020b). The similar patterns of upregulation of these transcription factors in two separate tissues suggests that these factors may play a key role in the transcriptional response to seawater entry, distinct from their typical association with circadian clock control (for discussion see West et al. 2020b). Potentially this history-independent aspect of the SV response to SW constitutes the primary response pathway, which then triggers the history-dependent downstream changes based on epigenetic differences transcription factor binding site access to other regions of the genome.

While work in the masu salmon has led to the suggestion that the SV may directly integrate photoperiodic information leading to coordination of physiological changes, we have been unable to detect expression of the key ingredients of this putative pathway in the SV transcriptome of the Atlantic salmon. Specifically, while Nakane et al. reported expression of the opsins *rh1*, *sws1*, *lws* and *opn4* in SVs of underyearling precocious male Masu salmon, no transcript expression for any of these genes was observed in our dataset. Nevertheless, our detection of five different opsin transcripts means that direct light sensitivity in the Atlantic salmon SV remains a possibility. Furthermore, levels of expression of *tshβa*, *tshβb,* the glycoprotein alpha subunit gene *glha1*, and of the thyrotropin receptor (*tshr*) were all undetectable in our analysis. While we could detect type 2 and type 3 deiodinase gene expression (*dio2a*, *dio2b* & *dio3*) in the SV, no effect of either photoperiodic history or salinity change was observed. Consistent with our data, a qPCR study reported photoperiodic insensitivity and low expression of *dio2a* and *dio2b* in the SV of Atlantic salmon smolts (Irachi et al. 2021). In contrast to our data, this study observed low *tshβa* and *tshβb* expression, although notably without photoperiodic change (Irachi et al. 2021). The previously reported transience of tshβb expression in the in the Atlantic salmon pituitary (Fleming et al. 2019) means it is possible that our study and that of Irachi et al. (2021) may have missed a peak expression of the TSH pathway key elements. Future research should collect SVs over a higher time resolution to be certain that transient effects are not missed. Taken together, however, our data suggests that photoperiodic influences on the SV are not tissue-intrinsic responses through an opsin-TSH-DIO cascade, and we therefore favour a model in which smoltification-related changes in SV function occur as secondary response to neuroendocrine responses to photoperiod mediated by the hypothalamo-pituitary axis. This may include classical changes in smoltification related hormones including cortisol, growth hormone and IGF1, the receptors for all of which are present in our RNAseq dataset (Supplementary Table 1).

While Nakane and colleagues have proposed that the SV is involved in seasonal reproductive regulation in masu salmon, there is no obvious link between the changes in the SV transcriptome observed in the present study and control of the gonadal axis in Atlantic salmon. Rather, several important aspects of our data are consistent with earlier work linking the SV to the regulation of CSF composition. The high contribution (> 15%) of transcripts for the ependymins, *epn1/2* to the overall transcriptomic signature is of particular interest. Ependymins are the dominant protein component of the CSF in teleosts, and their solubility is calcium-dependent (Hoffmann and Schwarz 1996; Shashoua 1991). Previous work highlights the high density of Ca^2+^ containing vesicles in the CSF-contacting globules of the SV coronet cells. These vesicles are thought to be physiologically controlled reservoirs that maintain calcium homeostasis in the cerebrospinal fluid (CSF) (Jansen et al. 1981, 1982; Graña et al. 2012; Cid et al. 2015). Our results suggest a revision of this hypothesis: the SV is a circumventricular organ responsible for high levels of secretion of ependymin to maintain CSF composition, and the vesicular packaging and solubility of ependymin depend on high levels of calcium in the secretory vesicles of the coronet cells. The extensive innervation of the SV by hypothalamic neuronal projections and by CSF-contacting dopaminergic neurons (Joy and Sathyanesan 1979; Sueiro et al. 2007), as well as the extensive transcriptomic responses of the SV to SW exposure would then reflect the need for precise control of ependymin secretion during the transition to a new aquatic environment.

Taken together, our data shows that in the Atlantic salmon, the SV transcriptome responds to SW exposure in a manner that depends on photoperiodic history and smoltification status. Significantly, most of the key elements of the opsin-TSH-DIO seasonal trigger pathway (e.g. *tshb* subunits, *glha1*, and *tshr*) are not present in our SV dataset, suggesting that in contrast to the proposed role of the SV in seasonal reproduction of masu salmon (Nakane et al. 2013), the Atlantic salmon SV does not play a coordinating role in smoltification. Available evidence is consistent with a role for the SV in regulation of CSF composition during transition between freshwater and marine environments. Regarding photoperiodic control of smoltification, we suggest that future work should focus on exploring the role of the hypothalamus and pituitary and whether pituitary derived tshβb plays a functional role in driving the smoltification.

## Supplementary Information

Below is the link to the electronic supplementary material.

Supplementary file1 (PDF 59 KB)

Supplementary file2 (XLSX 15494 KB)

Supplementary file3 (XLSX 239 KB)

Supplementary file4 (XLSX 297 KB)

Supplementary file5 (XLSX 196 KB)

Supplementary file6 (XLSX 211 KB)

Supplementary file7 (XLSX 227 KB)

Supplementary file8 (XLSX 10 KB)

Supplementary file9 (XLSX 14 KB)

## Data Availability

Data is accessible online (GEO accession number GSE291612).
