# Supplementary figures and images for: Photoperiodic history modulates the response of the saccus vasculosus transcriptome to seawater exposure in Atlantic salmon
Source: J Comp Physiol A Neuroethol Sens Neural Behav Physiol. 2025 Nov 6;212(1):69–78. doi: 10.1007/s00359-025-01779-w (PMC13038667; doi:10.1007/s00359-025-01779-w)

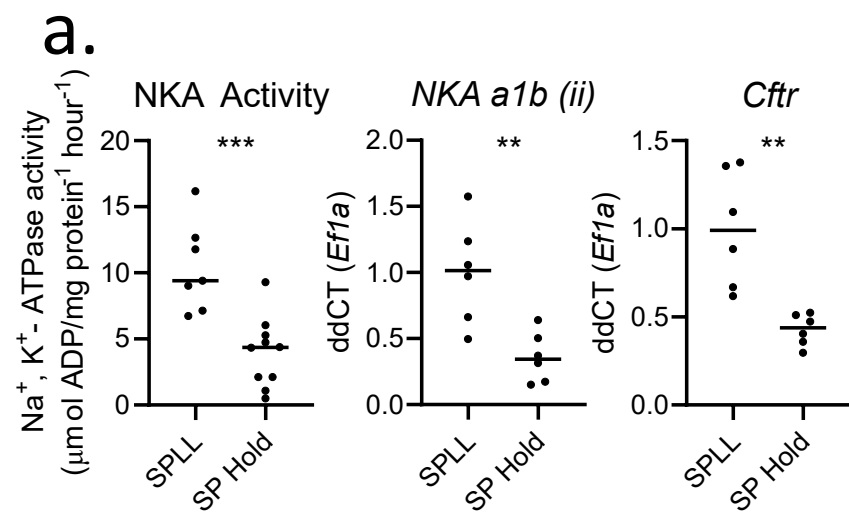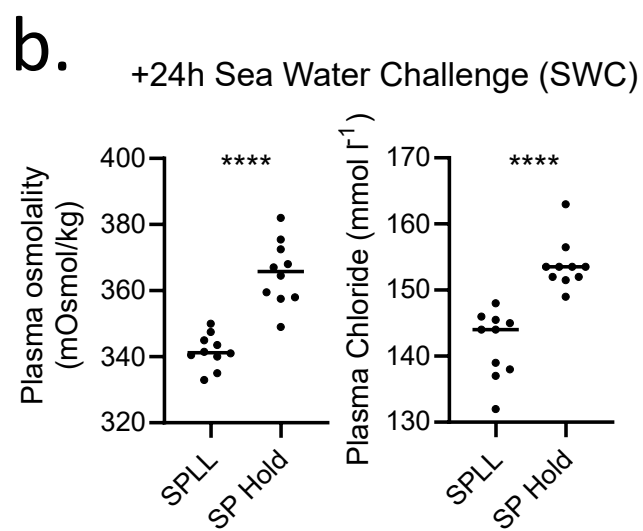

Supplement: Supplementary file 1 — Supplementary file1 (PDF 59 KB) [file 359_2025_1779_MOESM1_ESM.pdf]
